# Intramuscular vaccination of mice with the human herpes simplex virus type-1(HSV-1) VC2 vaccine, but not its parental strain HSV-1(F) confers full protection against lethal ocular HSV-1 (McKrae) pathogenesis

**DOI:** 10.1371/journal.pone.0228252

**Published:** 2020-02-06

**Authors:** Shan K. Naidu, Rafiq Nabi, Nagarjuna R. Cheemarla, Brent A. Stanfield, Paul J. Rider, Nithya Jambunathan, Vladimir N. Chouljenko, Renee Carter, Fabio Del Piero, Ingeborg Langohr, Konstantin G. Kousoulas

**Affiliations:** 1 Division of Biotechnology and Molecular Medicine, Louisiana State University, Baton Rouge, Louisiana, United States of America; 2 Department of Pathobiological Sciences, Louisiana State University, Baton Rouge, Louisiana, United States of America; 3 Department of Molecular Genetics and Microbiology, Duke University School of Medicine, Durham, North Carolina, United States of America; 4 Department of Veterinary Clinical Sciences, School of Veterinary Medicine, Louisiana State University, Baton Rouge, Louisiana, United States of America; Wayne State University School of Medicine, UNITED STATES

## Abstract

Herpes simplex virus type-1 (HSV-1) can cause severe ocular infection and blindness. We have previously shown that the HSV-1 VC2 vaccine strain is protective in mice and guinea pigs against genital herpes infection following vaginal challenge with HSV-1 or HSV-2. In this study, we evaluated the efficacy of VC2 intramuscular vaccination in mice against herpetic keratitis following ocular challenge with lethal human clinical strain HSV-1(McKrae). VC2 vaccination in mice produced superior protection and morbidity control in comparison to its parental strain HSV-1(F). Specifically, after HSV-1(McKrae) ocular challenge, all VC2 vaccinated- mice survived, while 30% of the HSV-1(F)- vaccinated and 100% of the mock-vaccinated mice died post challenge. VC2-vaccinated mice did not exhibit any symptoms of ocular infection and completely recovered from initial conjunctivitis. In contrast, HSV-1(F)-vaccinated mice developed time-dependent progressive keratitis characterized by corneal opacification, while mock-vaccinated animals exhibited more severe stromal keratitis characterized by immune cell infiltration and neovascularization in corneal stroma with corneal opacification. Cornea in VC2-immunized mice exhibited significantly increased infiltration of CD3^+^ T lymphocytes and decreased infiltration of Iba1+ macrophages in comparison to mock- or HSV-1(F)-vaccinated groups. VC2 immunization produced higher virus neutralization titers than HSV-1(F) post challenge. Furthermore, VC-vaccination significantly increased the CD4 T central memory (TCM) subsets and CD8 T effector memory (TEM) subsets in the draining lymph nodes following ocular HSV-1 (McKrae) challenge, then mock- or HSV-1(F)-vaccination. These results indicate that VC2 vaccination produces a protective immune response at the site of challenge to protect against HSV-1-induced ocular pathogenesis.

## Introduction

Herpes Simplex Virus (HSV) -1 and -2 are highly prevalent human pathogens. Commonly, virus replication initiates in epithelial cells and can establish latency in innervating sensory neurons. These viruses may reactivate periodically producing localized lesions in facial and genital epithelial tissues[1]. It has been estimated that 67% and 11% of world population are infected with HSV-1 and HSV-2, respectively [2, 3], while 33% of the population is estimated to have a latent infection with HSV-1[4].

Treatment of HSV infections includes primarily systemic administration of the antiviral compounds acyclovir, valacyclovir and famciclovir, while trifluridine and ganciclovir gels are being used for topical treatment [5]. HSV can cause a spectrum of disease including but not limited to primary and recurrent infections of mucous membranes such as gingivostomatitis, herpes labialis, and genital infections. They can also cause neonatal and congenital HSV infection, visceral HSV infections in immunocompromised hosts, and HSV encephalitis[6]. Ocular complications include lid, conjunctival, corneal, intraocular infections, and retinitis [5–8]. Although HSV-2 is more restricted to infection of the genital epithelium [9], HSV-1 can cause infection on both genital and ocular areas [5, 9]. HSV-1 is able to establish a latent infection in trigeminal ganglion and spinal(dorsal) ganglia [10], which upon reactivation can cause severe ocular infection. The major cause of infectious blindness in many developed countries is herpes infection and associated immunopathogenesis [11].

Although a significant number of people are already infected with HSV, epidemiological studies in maternal HSV transmission indicate that pre-existing immunity may reduce the number of newly transmitted infections and associated pathological consequences. Specifically, the rate of viral transmission in pregnant mothers is higher for primary than recurrent infection [12]. Furthermore, primary infections in the third trimester of pregnancy have a higher transmission rate compared to the first trimester [13, 14], probably due to the shorter span of time for establishment of anti-viral immune responses. These and other studies strongly suggest that vaccine mediated immunity may lower the risk of acquisition and transmission.

Although a number of vaccine approaches are being currently investigated, currently there is no HSV vaccine that has been approved by FDA for human use. Current approaches include subunit, multivalent and live vaccine with partial or complete deletion of HSV proteins [15–19]. Because there is a significant homology between HSV-1 and HSV-2 [20], a vaccine that generates cross reactive immunity may have significant benefit over a type-specific vaccine. We believe a successful HSV vaccine should be able to provide protection from both ocular and genital herpes acquisition. In addition to prophylactic action, a therapeutic vaccine may suppress viral reactivation from TG and/or quickly neutralize reactivating virus, minimizing the risk for HSV- related ocular pathogenesis in already infected individuals.

Generally, live attenuated vaccines induce stronger immunity against a specific pathogen compared to a subunit vaccine [21]. Recently, we developed a live attenuated HSV-1 vaccine which is safe exhibiting strong immune responses against both HSV-1 and HSV-2 genital infection in both murine model and guinea pig models [22, 23]. The vaccine is based on the live-attenuated HSV-1 strain, named VC2, which has partial deletions in the viral glycoprotein gK(31-68aa) and the UL20(4-22aa) membrane protein. Previously, we showed that gK is involved in facilitating fusion of the viral envelope with cellular membranes during virus entry [24–28]. Importantly, we showed that gK (31-68aa) deletion prevented the virus from entering axonal termini of neurons in culture and prevented retrograde virion transport after ocular infection [29]. UL20 physically interacts with gK and these interactions are necessary for intracellular transport and functions of both g K and UL20 [30]. Both gK and UL20 interact with viral glycoprotein B, which is the sole fusogen enabling the virus to enter via fusion of the viral envelope with cellular membranes, as well as induce cell-to-cell fusion allowing the virus to spread from cell-to-cell [28, 30–35]. Intramuscular vaccination of mice with VC2 resulted in complete protection from lethal HSV-1 and HSV-2 challenge via the vaginal route [22] indicating that VC2 generated cross-reactive immunity to both HSV-1 and HSV-2 infections.

Herein, we report that intramuscular immunization of live attenuated VC2 provided complete protection against lethal ocular challenge in a murine model of HSV-1 infection using the HSV-1 strain McKrae as the challenge virus. Our findings suggest that VC2 immunization induces a significantly improved immune response compared to its parental HSV-1(F) wild-type strain, although they both protect the mice from lethal challenge, characterized by increased production of neutralizing antibodies, alteration of cellular lymphocyte infiltration and prevention of pathogenesis.

## Materials and methods

### Construction of attenuated virus

The construction of VC2 recombinant virus and its use a live-attenuated vaccine was described previously [22, 36, 37].

### Study animals

Specific-pathogen-free female SCID and mice were obtained from Charles River Laboratories (Wilmington, MA). and used at approximately 8 weeks of age. To evaluate vaccine efficacy generated by VC2 vaccine compared to wild type virus F BALB/C mice were used as experimental model. For these studies, female 8- to 12-week-old mice were purchased from Jackson Laboratories (Bar Harbor, ME). Each mouse was identified with an ear tag (National Band and Tag Company, KY, USA). All animal experiments were carefully reviewed and approved by the Louisiana State University Institutional Animal Care and Use Committee and adhered to the Public Health Service Policy on the Humane Care and Use of Laboratory Animals, the National Research Council Guide for the Care and Use of Laboratory Animals, and the United States Department of Agriculture (USDA) Animal Welfare Regulations. LSU is licensed as an animal research facility by the USDA and has an Animal Welfare Assurance on file with the Office of Laboratory Animal Welfare. The animal care and use program at LSU is accredited by the Association for Assessment and Accreditation of Laboratory Animal Care International (AAALAC).

### Vaccination protocol

Mice were mildly anesthetized using inhalation of 2–3% isoflurane before intramuscular vaccination. Mice were divided into three groups to receive either VC2 vaccine or HSV-1 (F) vaccine or mock inoculations. Two intramuscular injections were administered at 3-weeks intervals with 1×10^7^ PFU in 100μl culture supernatant. Mock vaccination group received uninfected cell culture supernatant. Mice were then observed daily for any clinical symptoms. The first intramuscular injection was given on left hind limb while second injection was given on right hind limb after 21 days from the first injection.

### Ocular challenge

Four weeks after the final dose of vaccination, mice were subjected to intraocular challenge in both eyes with 2 x 10^4^ plaque forming units of HSV-1 strain McKrae in 2 μL of culture medium (DMEM containing Primocin) using a micropipette. Mice were anesthetized, eyes were dried with cotton swab and culture medium containing virus were applied on the ocular surface. Following challenge, mice were daily observed for any clinical symptoms.

### Clinical scoring of ocular disease

Clinical ocular evaluation was performed by a veterinary ophthalmologist using a handheld slit lamp biomicroscope on days 2, 8 and 16 post challenge. Corneal opacification results in a reduction in corneal clarity, discoloration of the corneal tissue as well as a reduced ability to view intraocular structures (such as the iris) through the lesion. Corneal opacification may be the manifestation of multiple pathologic tissue responses including corneal ulceration, cellular infiltration, corneal edema and finally, scar tissue formation or fibrosis. The corneal neovascularization present clinically were small mid-stromal corneal vessels that typically originated from the limbus. The ocular herpes and keratitis was clinically scored on a scale of 0 to 5: 0, Normal; 1, Conjunctivitis; 2, Conjunctivitis and Mild Keratitis (1+ corneal opacification); 3, Conjunctivitis and Moderate Keratitis (2+ corneal opacification and corneal vascularization developing); 4, Moderate Blepharoconjunctivitis and Keratitis (3+ corneal opacification and corneal vascularization); 5, Severe Blepharoconjuctivitis and Keratitis (4+ corneal opacification and vascularization), corneal perforation was often present.

### Blood collection

300–500 μL of blood per animal were collected from facial vein on both day 21-post final vaccination and day 5- post challenge on 10 mice from each group under the isoflurane anesthesia. Blood was allowed to clot at 4°C overnight in 5mL falcon tubes (Becton Dickinson, Franklin lakes, NJ) before separating serum by centrifugation in a 2-mL Sarstedt Screw Cap Micro Tubes (Sarstedt Inc., Newton, NC). Separated serum werestored at −20°C until analysis.

### Tissue collection and evaluation of neurovirulence

Terminal euthanasia were scheduled on 3 dpi and 15 dpi for histopathological evaluation of cornea. Mice were euthanized using carbon dioxide, followed by cervical dislocation prior to necropsy procedure. The cornea, mandibular lymph node, spleen and other tissues were preserved either in 10% neutral buffered formalin or snap frozen in liquid nitrogen and stored at −80 °C until further analysis. Trigeminal ganglion was excised under dissecting microscope. Total tissue DNA was extracted using Qiagen DNeasy Blood and Tissue Kit (Qiagen) and viral genomes were estimated using quantitative PCR.

To determine the sensitivity of the qPCR assay, HSV-1 and HSV-2 genomic DNA were quantified and their respective molar concentrations was calculated using the formula:–{μgDNA × (pmol/660) × (106 pg/1μg) × (1/N) = pmol DNA, where N = number of nucleotides}. Ten-fold serial dilutions ranging from 105–100.1 molecules were used as template samples in Taqman PCR reactions, and water was used a no template control. qPCR was performed on the Applied biosystems 7900HT Fast Real-Time PCR System. HSV target DNA was detected at the lowest dilution (2.7×10−8 μg of DNA) containing 3 copies per μl. No viral DNA was detected in the no template control sample. The linear range of detection ranged from 3 to 10^6^ viral DNA copies with a mock-vaccinated mice exhibiting more than 10^6^ viral DNA copies per sample.

### ELISA for HSV specific antibody

Serum from all groups of mice were collected 3 weeks after booster vaccination and 5 days after ocular challenge infection. To determine the antigen specific antibody isotypes, 96-well plates (high protein binding plates; Corning, Inc) were prepared by coating with 50μL of HSV-1 McKrae infected cell lysate (20 μg/ml) at 4’C overnight. Then plates were washed and blocked with 5% non-fat dry milk in PBS for 2 hours at room temperature. Then 1:100 diluted serum from each group of mice were added to each well and incubated for 2 hours at room temperature, followed by incubation with Goat Anti-Mouse HRP conjugated antibodies. The TMB substrate solution was used to develop color reaction and optical density of each well will be quantified with a plate reader.

### Detection of infectious virus on corneal tear film

Mouse tears were collected by gently swabbing the external surface of cornea with sterile swabs at day 2, 8, and 16-post challenge. The corneal swabs were transferred collection tubes containing 1 ml of culture medium and frozen at -80’C until use. The swab culture was added to confluent Vero cells for 60 min at 37°C on rocker, and then overlaid by 1% methylcellulose before incubating the plates for 72 hrs. The plaque forming units were quantified after fixing the cells with formalin and staining with crystal violet. Each plaque forming units (PFU) is representative of an infectious virus.

### Neutralization assay

Neutralization assays were performed with sera collected at 3 weeks after primary and 3 weeks after booster vaccination from immunized mice. Sera (1:40 dilution) from immunized mice were treated at 56’C for 30 min to heat inactivate the complement. HSV-1 McKrae virus adjusted to 100 PFU per each ml of diluted serum for 1 h at 37°C. Then confluent vero cells in 6 well plates were inoculated with the virus-serum mixture and neutralization activity was determined by plaque assay [38]. The neutralization activity was measured as percentage reduction in the plaque forming units compared to that of serum from non-immunized mice (control).

### Flow cytometry

Spleen were collected from immunized mice on day 3-post challenge. Spleen were sliced and dissociated into single cell suspension in complete cell culture medium and allowed to go through 70 um-pore size mesh filter. Cells were labeled with antibodies for 30 min at 4°C and washed with PBS/1% BSA before fixing them with 1% paraformaldehyde. Cells were analyzed using a FACScan flow cytometer (BD Biosciences, San Jose, CA, USA) and FlowJo software (version 7.6.3; Tree Star, Ashland, OR, USA). The following BD antibody panels were used for flow cytometry: T lymphocyte panel with CD3e PerCP (cat. no. 561089), CD4 APC (cat. no. 553051), CD8 Alexa Fluor 488 (cat. no. 557668); CD4 memory cell panel with CD4 FITC (cat. no. 553729), CD44 PerCP cy5.5 (cat. no. 560570), CD62L APC (cat. no. 561919); and CD8 memory cell panel with CD8a Alexa Fluor 488 (cat. no. 557668), CD44 PerCP cy5.5 (cat. no. 560570) and CD62L APC (cat. no. 561919).

### Histopathology and immunohistochemistry

Corneal samples were fixed in 10% formalin, dehydrated through increasing ethanol gradients and paraffin embedded to prepare 4-μm sections. Standard hematoxylin and eosin (HE) staining and Masson’s Trichrome Staining was carried out in LSU histopathology core facility. Immunohistochemical staining was performed on paraffin-embedded sections against HSV-1 antibody (rabbit anti HSV-1 polyclonal), Iba1, CD3 and CD45R. HSV-1 virus was detected using an antibody. Finally, the slides were visualized using a light microscope (Nikon, Japan) and pictured using Olympus DP72 camera. Quantification of immune cell infiltration (Iba1, CD3 and CD45R positive cells) in corneal tissue sections were performed on the IHC-stained slides. Total number of cells from 10 randomly selected regions per cornea were quantified.

### Statistical analysis

Statistical significance was calculated by unpaired t test and one-way ANOVA or two-way ANOVA (when necessary) to determine the differences between the animal groups, followed by a Tukey-Kramer test to correct for multiple comparisons using Graph Pad InStat 3 (GraphPad Software, La Jolla, CA, USA).

## Results

### Safety study

**Fig 1:**
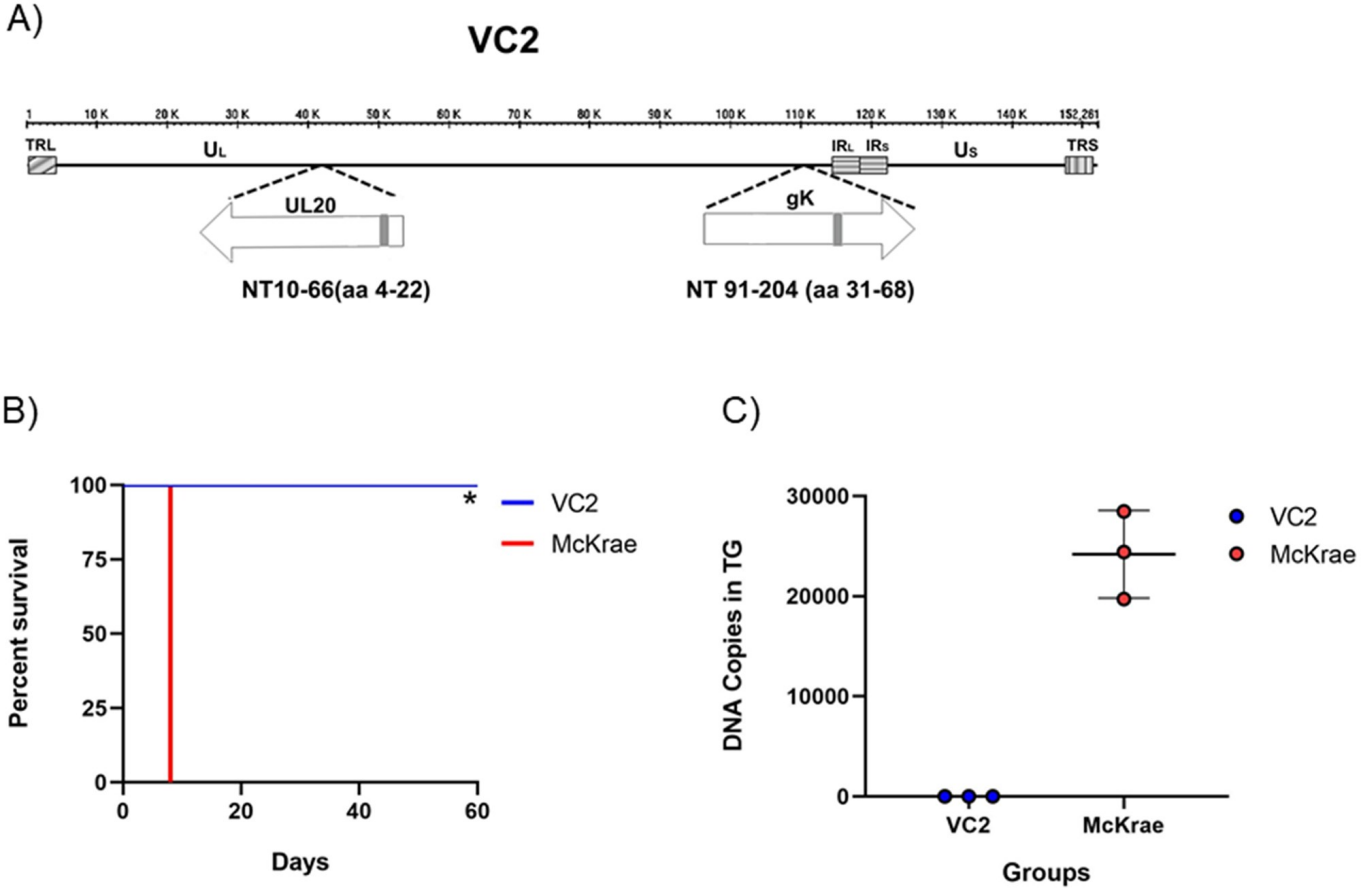
Safety evaluation of VC2 vaccine. (A) A schematic diagram of the genomic arrangement in the VC2 mutant vaccine strain. (B) Kaplan-Meier survival curve in SCID mice following intramuscular injection of 10^6^ PFU of VC2, or HSV-1(McKrae) virus. (C) DNA copy number in trigeminal ganglia of VC2 or McKrae infected mice. N = 3 per group. Data are represented as Mean ± SEM. * P ≤ 0.05 by Gehan-Breslow-Wilcoxon test.

The live-attenuated VC2 vaccine strain was constructed by deleting amino terminal domains of UL20 (4-22aa) and gK (31-68aa) [22](Fig 1A). This attenuated virus was shown to be an effective vaccine in mice and guinea pigs, without producing any observable adverse reactions [23, 36, 37, 39, 40]. To further test whether VC2 may cause any adverse effects in immunocompromised animals additional toxicity studies were performed using SCID mice. Three mice in each group were vaccinated intramuscularly with either VC2 or HSV-1(McKrae) using 10^6^ PFU. VC2-infectted mice did not exhibit any adverse reactions and were euthanized on Day 60 post vaccination, while all animals inoculated with HSV-1(McKrae) exhibited neurotological signs as early as few days post infection and died by day 10 (Fig 1b). Quantitative PCR (qPCR) revealed that trigeminal ganglia of the VC2-vaccinated mice had no detectable viral DNA at day 60 post immunization, while the McKrae-vaccined mice HSV-1 McKrae produced high copy numbers of HSV-1 DNA at day 10 post immunization (Fig 1C).

### VC2 vaccination elicits protection against HSV-1(McKrae) lethal ocular challenge

**Fig 2:**
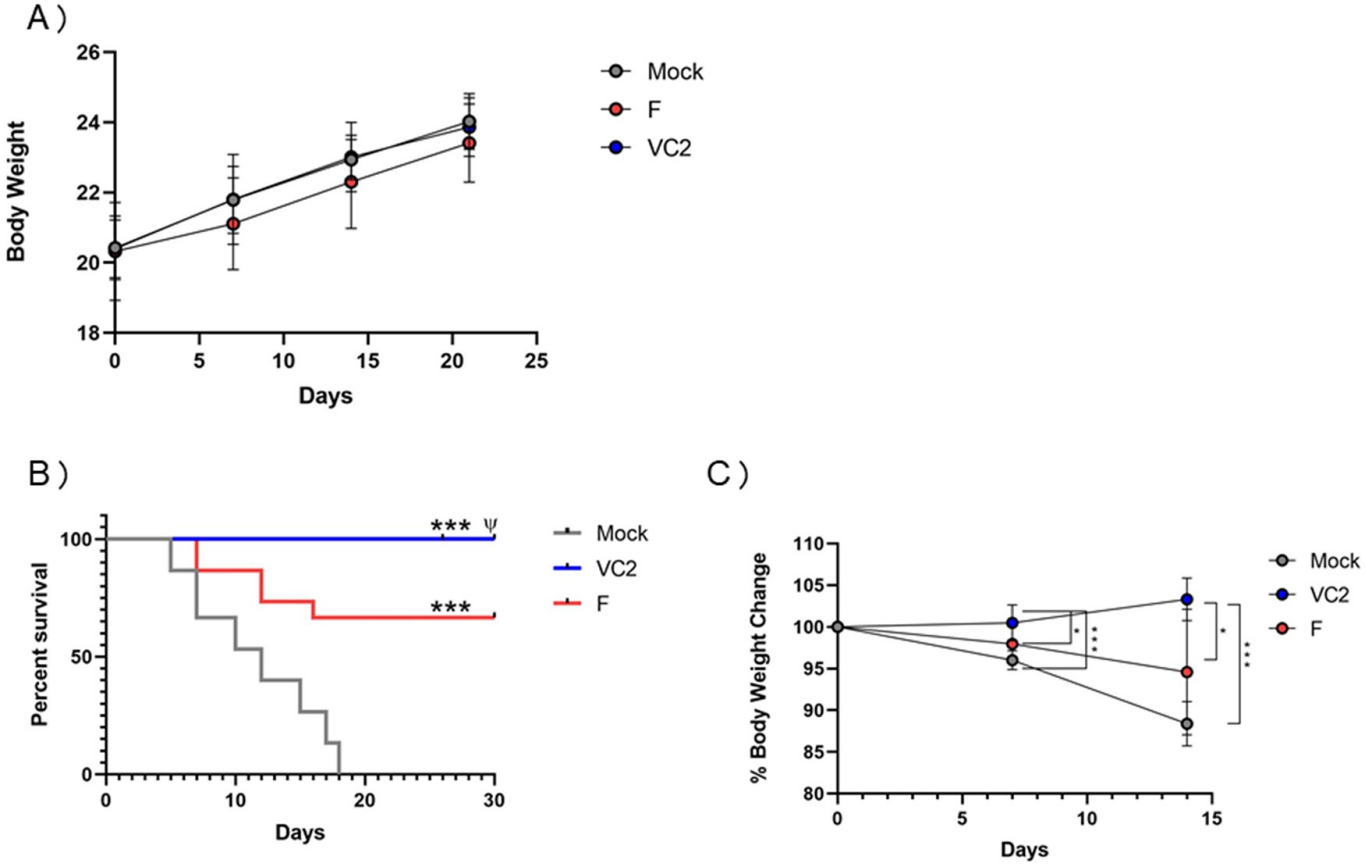
Effect of VC2 vaccination on body weight and survival in BALB/C mice. A) Body weight following immunization is presented as percentage change. Ocular challenge model: Animals were vaccinated twice 21 days apart followed by ocular challenge with 1x10^4^ HSV-1 (McKrae) 3 weeks after last vaccination and monitored for 25 days. N = 15 mice per group. B) Kaplan-Meier Survival analysis following ocular challenge. (C) Post-challenge body weight on day 0, 7 and 15. Percentage change in weights post challenge were presented in all three groups and percentages were normalized to the initial weight at day 0. Data are represented as Mean ± SEM. Statistical analysis was done using One-Way ANOVA and Tukey-Kramer test. *P ≤ 0.05 **P ≤ 0.01, ***P ≤ 0.001.

To test the VC2 immunization efficacy against lethal HSV-1 ocular challenge, a prime boost vaccination strategy was utilized followed by ocular infection with HSV-1(McKrae). Eight to 10 weeks Balb/C mice were vaccinated with 1×10^7^ PFU of either VC2 or its parental virus HSV-1 (F) at three week-intervals via the intramuscular route. Control groups were mock-vaccinated with cell culture supernatant (equal volumes). All three groups of mice had similar body weight after vaccination indicating that there was no adverse effect due to vaccination (Fig 2A). All groups were intraocularly challenged (both eyes) at 21 days post vaccination with 2 x 10^4^ PFU of the HSV-1 strain McKrae(Fig 2B). Following ocular challenge, the mice were monitored daily for signs of ocular disease. All VC2 vaccinated-mice survived, while 30% of the HSV-1(F)- vaccinated mice died between day 6 and 17 post challenge. VC2-vaccinated mice did not lose any weight, while both mock and HSV-1(F)- vaccinated groups exhibited significant reductions in body weight compared to the VC2-vaccinated group (Fig 2C).

### Effect of vaccination on ocular disease and virus shedding

**Fig 3:**
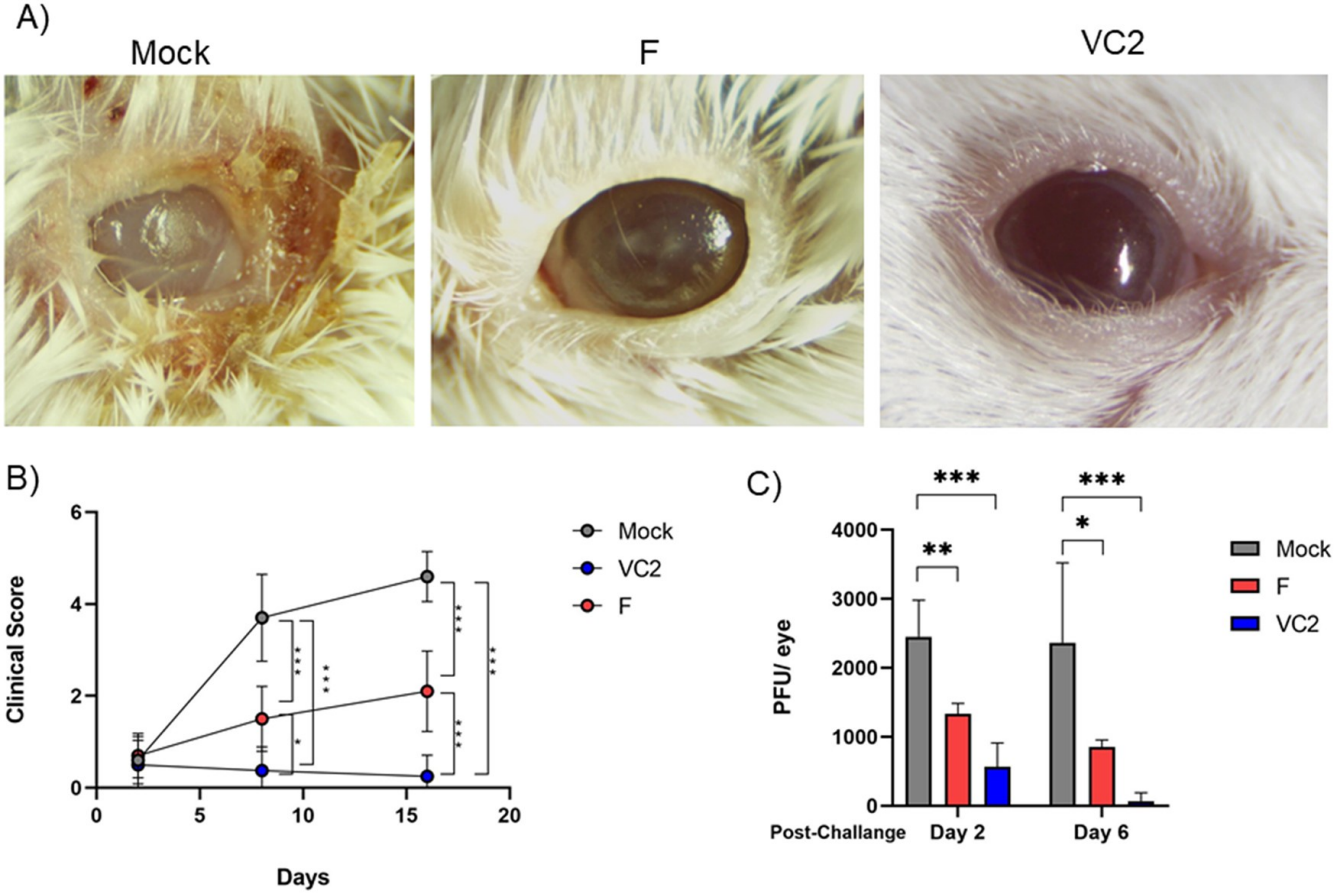
Clinical characterization of eyes after HSV-1 ocular challenge. (A) Representative clinical image of mock-, F- and VC2-vaccinated mice. (B) Clinical scoring of herpetic stromal keratitis in eyes after HSV-1 (McKrae)-ocular challenge. Eyes were examined by slit lamp biomicroscopy on days 2, 8 and 16 post challenge and scored for blepharitis, corneal opacity, corneal scarring, and neovascularization. calculated using One-way ANOVA. (C) Ocular viral shedding on 2 dpi and 6 dpi, presented as plaque forming units (PFU) per eye. Statistical analysis was done using One-Way ANOVA and Tukey-Kramer test. *P ≤ 0.05 **P ≤ 0.01, ***P ≤ 0.001.

Following ocular challenge, we evaluated ocular opacification by slit lamp microscopy and clinical scores were recorded until day 16 post challenge. Mice were scored on a scale of 0–5 (0, normal; 1, conjunctivitis; 2, conjunctivitis with corneal opacification; 3, conjunctivitis with corneal opacification and corneal vascularization developing; 4, moderate blepharoconjunctivitis with corneal opacification and corneal vacularization; 5, severe blepharoconjuctivitis, corneal opacification, vascularization and often corneal perforation. A marked chronic keratitis was observed in mock-vaccinated animals characterized by blepharoconjunctivitis, corneal opacification and neovascularization and a time-dependent increase in severity of disease was noted (Fig 3A and 3B). VC2-vaccinated mice did not exhibit any ocular keratitis and completely recovered from minimal initial conjunctivitis observed immediately after ocular challenge (Fig 3A, right panel). In contrast, HSV-1(F)-vaccinated mice developed time-dependent progressive keratitis characterized by the development of multiple corneal opacification foci Fig 3A, middle panel panel).

Ocular virus shedding was assessed during the first week following challenge with McKrae. Compared to HSV-1(F) F-vaccination, VC2 vaccinated mice showed a significant reduction in virus shedding, with almost no virus recovered from the ocular swabs by day 6 post challenge. On the other hand, HSV-1(F) vaccination significantly lowered ocular viral shedding, but virus shedding persisted in tear film until day 6 post-infection (Fig 3C).

### Microscopic evaluation of ocular tissue

**Fig 4:**
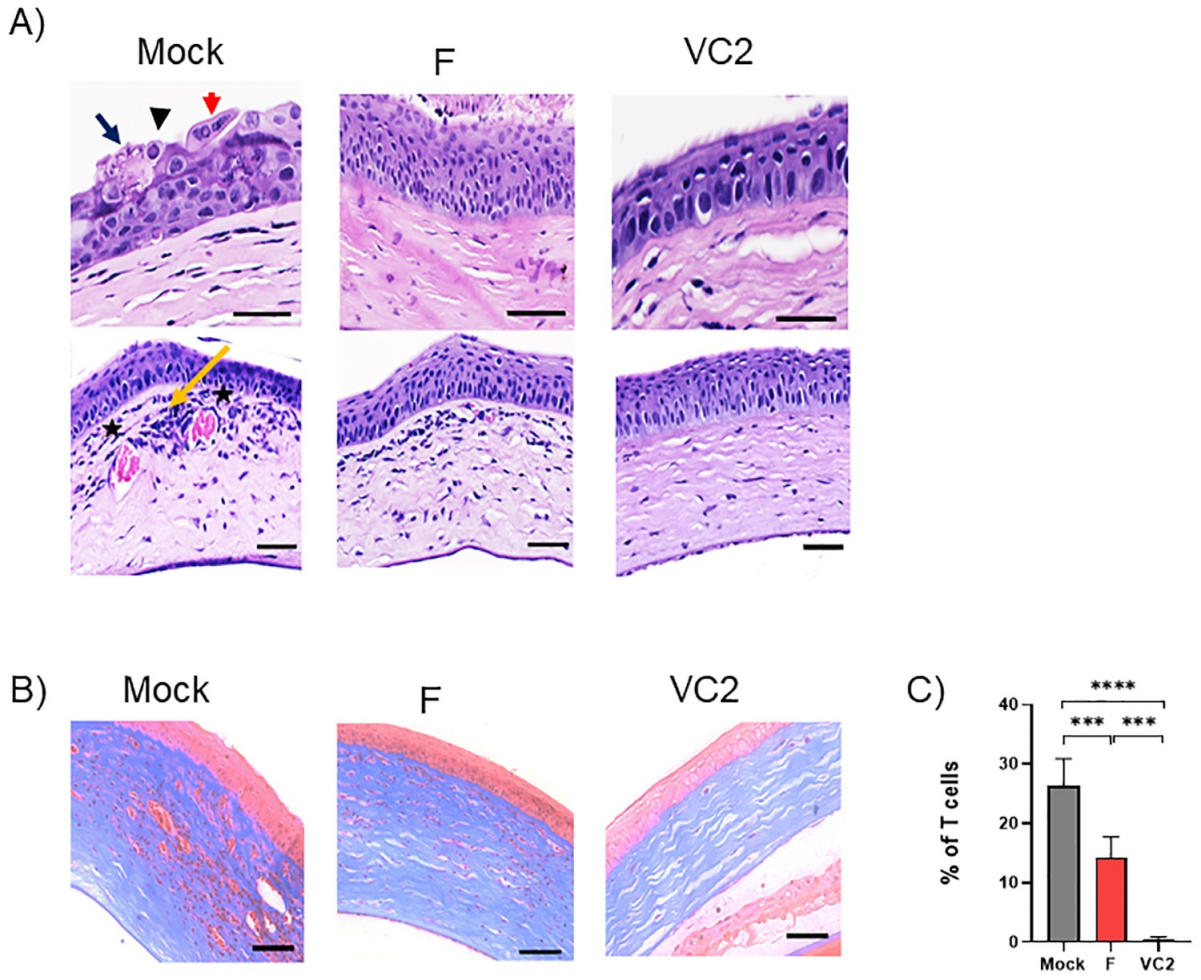
Histopathological characterization of corneas after HSV-1 ocular challenge. Hematoxylin & Eosin (H&E) stained images of corneas from 3 days post challenge (Top) 15 days post challenge (Bottom). Syncytial cells in the superficial corneal epithelium were shown in red arrow, eosinophilic intranuclear viral inclusions in black arrowhead, lytic epithelial lesions in black arrow, neovascularization were shown in * and inflammatory cells in yellow arrow. (B) Masson’s Trichrome staining of corneal tissue sections at 15 days post challenge. Blue stained area was extracellular matrix (collagen). Scale bar = 50 μm. (C) Quantification of fibrosis by from trichrome staining.

**Fig 5:**
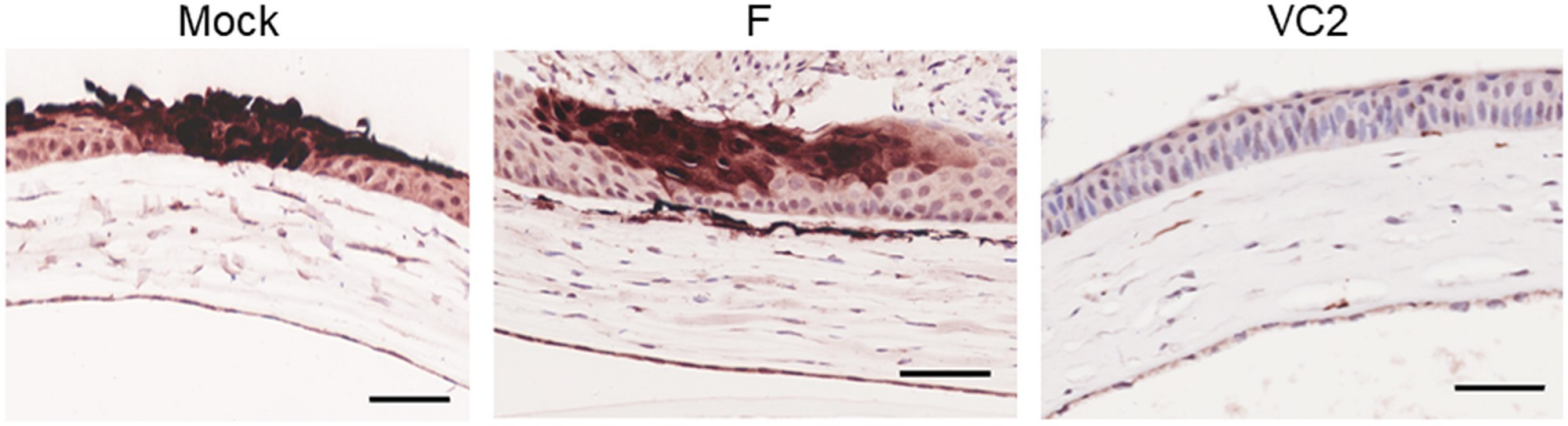
Immunohistochemical detection of HSV-1 viral antigen in cornea. Cornea sections were stained for presence of HSV-1 antigen 3 days post challenge in mock- F- or VC2- vaccinated mice. Brown stained areas were positive for HSV-1 viral protein. Scale bar = 50 μm.

Histopathological evaluation of cornea at day 3 post-challenge with HSV-1 (McKrae) in mock vaccinated animals showed development of acute keratitis characterized by multinucleated syncytial cells, eosinophilic intranuclear inclusions, and lytic epithelial lesions (Fig 4A). In contrast, there were no apparent lytic lesions or formation of syncytial cells in mouse corneas at 3 days post challenge in both VC2- and HSV-1(F)-vaccinated mice. Histopathological evaluation of corneas at day 15 post-challenge revealed significant disease progression to marked stromal keratitis characterized by abundant accumulation of mononuclear cells in corneal stroma in the mock (medium)-infected mice and neovascularization characterized by multiple newly formed large vascular channels lined by cuboidal endothelial cells (Fig 4A, bottom). The VC2-vaccinated animals did not develop any microscopic features of corneal disease. In contrast, the HSV-1(F)-vaccinated animals developed mild stromal keratitis with moderate numbers of infiltration of mononuclear cells and minimal stroma neovascularization. These results were further confirmed by trichrome staining, which revealed increased extracellular matrix deposition which surrounding the immune cell infiltrates in the corneal stroma of both mock (medium)- and HSV-1(F)-vaccinated animals (Fig 4B and 4C). Immunohistochemical staining with anti-HSV-1 polyclonal antibody revealed that the corneal epithelium was productively infected with virus spreading along the epithelial layers of cornea in both mock- and HSV-1(F)-vaccinated animals at day 3 post challenge (Fig 5). In contrast, the HSV-1 strain McKrae failed to spread across the corneal epithelium in VC2-vaccined mice.

### Immunohistochemistry of mouse ocular tissues

**Fig 6:**
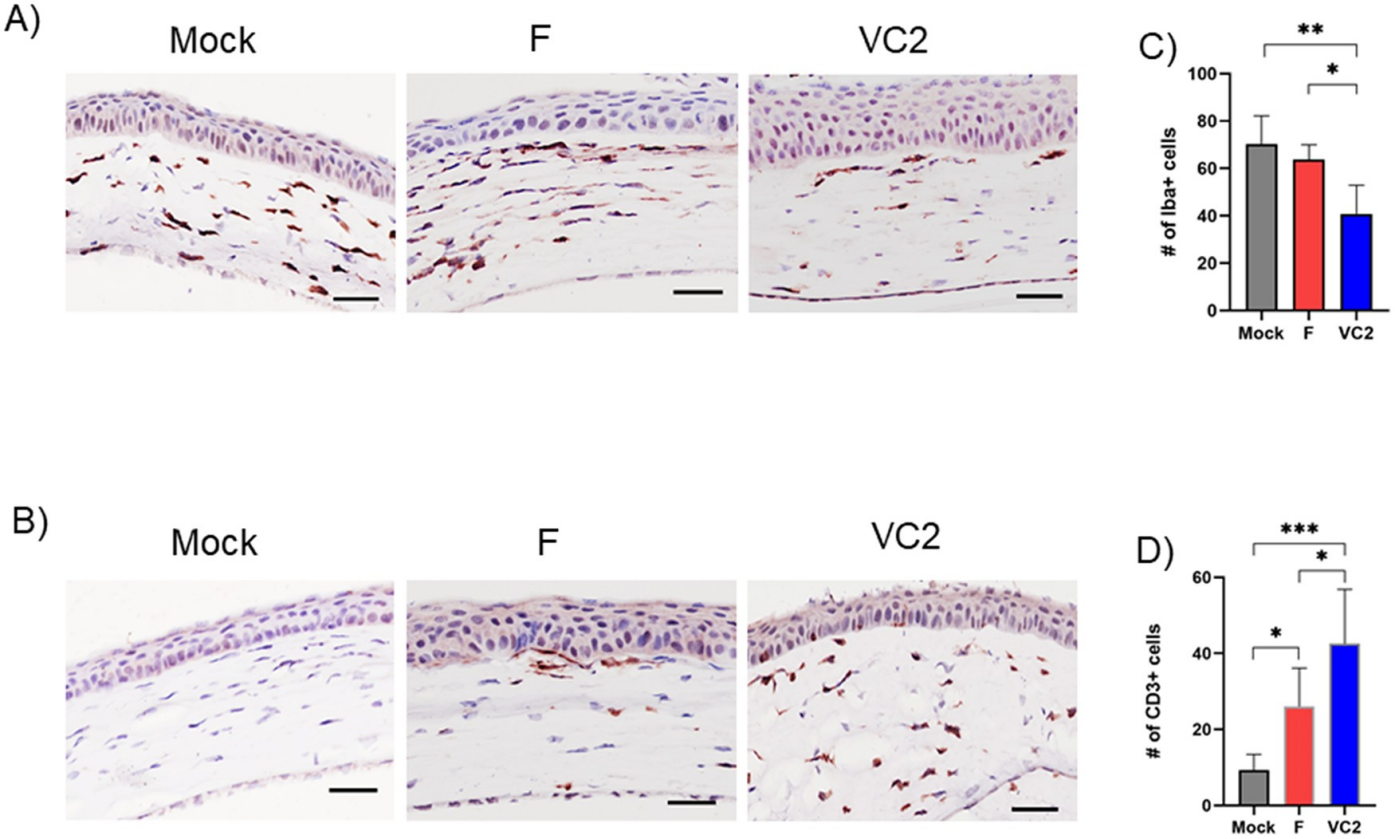
Characterization of immune cell infiltration in corneal stroma. (A) Corneal sections from 3 days post challenge were stained with (A) Iba1 for macrophage, and (B) CD3 for T lymphocytes. Quantification of (C) Iba1-positive macrophages, and (D) CD3-positive T cells in corneal tissue sections was performed from 10 different regions of cornea stroma per eye (n = 6). Statistical analysis was done using One-Way ANOVA and Tukey-Kramer test. *P ≤ 0.05 **P ≤ 0.01, ***P ≤ 0.001.

To determine relevant immune responses at the site of viral challenge in the vaccinated mice, immunohistochemical analysis was performed on the corneal tissue sections collected at day 3 post challenge (Fig 6). Corneal tissue sections were stained for macrophage (IBA1+) and T cell (CD3+). VC2-immunized mice exhibited significantly increased infiltration of CD3^+^ T lymphocytes (Fig 6B and 6D), and decreased infiltration of IBA1+ macrophages relative to those of mock- or HSV-1(F)-vaccinated groups (Fig 6A and 6C).

### Pre- and post-challenge adaptive immune responses

**Fig 7:**
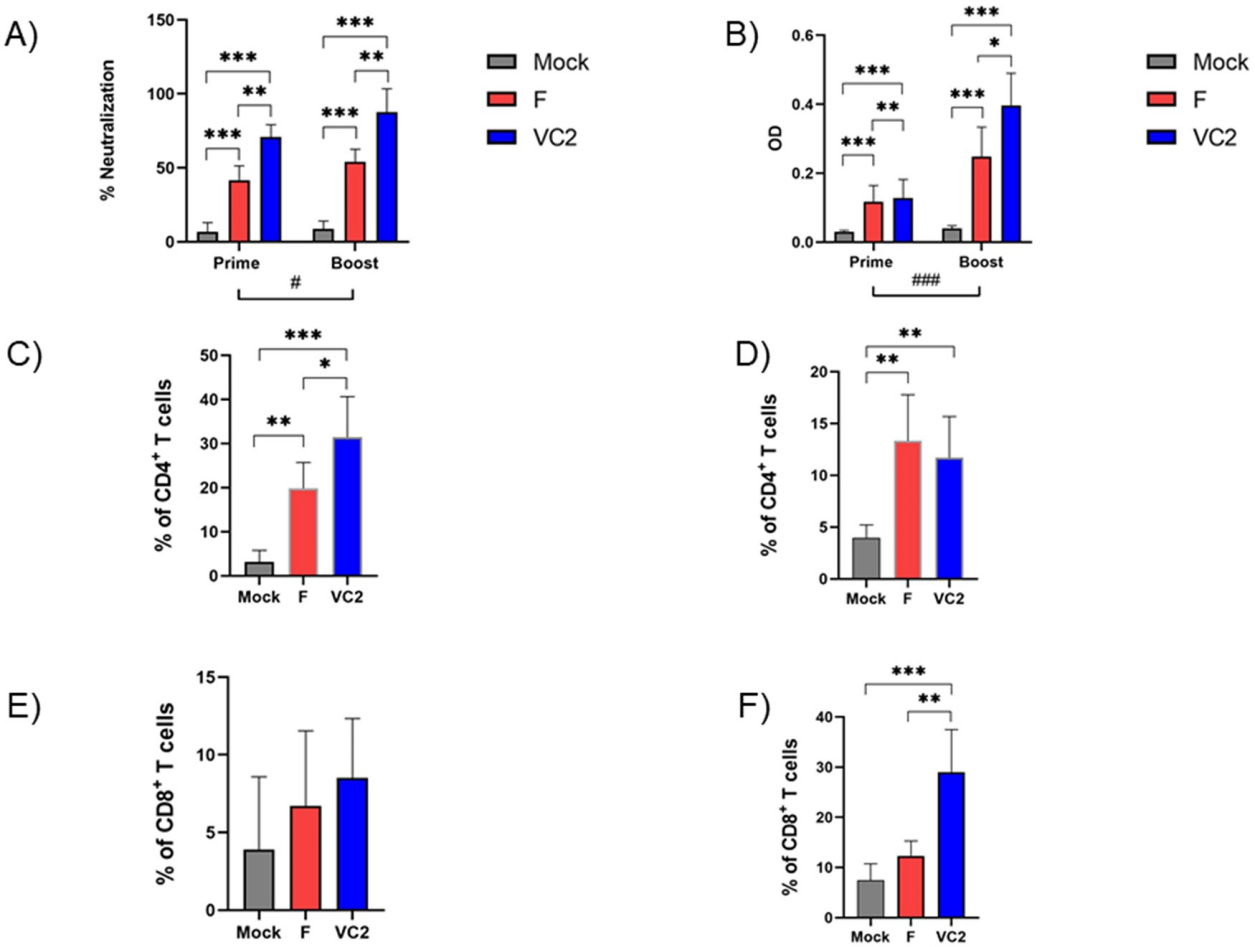
Analysis of systemic humoral and T-memory cell responses. (A) Plaque reduction neutralization assay at 1:40 dilution. (B) Indirect ELISA assay for HSV-1-specific IgG response at 1:100 dilution. Flow cytometric analysis of draining lymph nodes was performed on Day 3 post challenge to quantify the memory cell response. CD4-Tcm (C) CD4-Tem (D), CD8-Tcm (E) CD8-Tem (F) memory cell subtypes were shown. Statistical analysis was done using Two-Way ANOVA (A, B) and One-Way ANOVA and Tukey-Kramer test (C-F). *P ≤ 0.05 **P ≤ 0.01, ***P ≤ 0.001.

The plaque reduction neutralization assay was performed to evaluate the presence of neutralizing antibodies in the sera collected at 3 weeks after primary vaccination and 3 weeks after booster vaccination. Sera from all groups of mice were individually tested at 1:40 dilution. Sera from mice immunized with VC2 exhibited higher virus-neutralizing activity after primary and booster vaccination in comparison to the mock-vaccinated mice or HS-1(F)-vaccinated mice (Fig 7a). Serum collected from animals at 3 weeks after booster vaccination and 5 days after ocular challenge were evaluated for HSV-1 specific IgG binding antibody. Using two-way ANOVA analysis there were significant differences between HSV-1(F) and VC2-vaccinated mice groups of mice pre-challenge, there was a significant difference between VC2 and F vaccinated groups post challenge (Fig 7b).

To evaluate cellular responses elicited by vaccination, draining lymph nodes of the immunized mice were obtained at 3 days post challenge and prepared as a single cell suspension. A gating strategy is shown in supplementary S1 Fig. Subsequently, phenotypic characterization based on the of expression of CD44 and CD62L into central memory (TCM; CD44^hi^ CD62L^hi^), and effector memory (TEM; CD44^hi^ CD62L^lo^) were evaluated. The draining lymph nodes of VC-2-vaccinated mice exhibited significantly increased CD4 TCM subsets and CD8 TEM subsets relative to those of HSV-1(F)-and mock-vaccinated mice (Fig 7C–7F).

## Discussion

Previously, we generated a novel mutant HSV-1 (VC2) with a partial deletion in both gK and UL20 and reported that intramuscular vaccination of VC2 was able to protect mice from lethal intravaginal HSV-1 and -2 challenge [41]. In addition, intramuscular immunization of guinea pigs with VC2 conferred significant protection against HSV-2 genital challenge [23]. The goal of the present study was to evaluate and compare the efficacy of VC2 compared to the wild type HSV-1(F) in the murine ocular model. Our data indicate that VC2 is a safe vaccine that is highly efficacious in protecting mice against ocular challenge with the HSV-1 strain McKrae.

Of particular interest to our studies was the potential differences between the VC2 live attenuated vaccine and its parental strain HSV-1(F). The latter is also partially attenuated in mice in comparison to clinical HSV strains such as the HSV-1(McKrae) virus isolated from an ocular human infection. Importantly, HSV-1(F) intramuscular vaccination partially protected mice from lethal McKrae challenge, but it was unable to control morbidity. In contrast, VC2 exhibited superior protection and morbidity control against HSV-1(McKrae) lethal challenge. gk 31–68 deleted virus has been shown to defective in entering mouse neurons[42] by fusion of the viral envelope with cellular membranes[29], while it can enter into Vero cells via endocytosis and replicate in similar titers to the parental virus. This substantial difference in virus entry may induce significant downstream signaling responses that result in the observed differential efficacy in vaccine-induced protection. This hypothesis is further supported by the fact that the presence of UL20 significant alters the downstream expression of inflammatory cytokines (not shown). In addition, VC2 is slightly fusogenic producing small syncytial in cell culture in comparison to HSV-1(F). This increase fusogenicity may also affect its ability to spread in tissues resulting in vaccine enhancement.

The absence of keratitis and lower viral shedding at day 3 days post challenge in VC2 vaccinated animals coincided with the absence of neovascularization and cellular infiltration. This result suggests that there might be immediate control of the viral replication following challenge in VC2- vaccinated mice in contrast to HSV-1(F) vaccinated mice. This hypothesis is supported by the presence of viral antigens in HSV-1(F) and mock-vaccinated mice at day 3 post infection, while no viral antigens were detectable in VC2 vaccinated mice. Thus, VC2 immunization generated virus specific immune responses that are significantly stronger and effective at controlling viral replication following McKrae ocular challenge. The involvement and protective role of T cells during HSV-1 infection is well established [43–46] with IFN-γ playing a pivotal role [47]. A significant increase of T cells (CD3+) was observed in VC2, but not HSV-1(F) or mock-vaccinated mice suggesting that these infiltrating T cells may play a role in antiviral protection. Within the T cell compartment, both CD4 and CD8 have been shown to be involved in conferring protection, as well as resultant immunopathogenesis with CD4 believed to represent a pro-inflammatory response [43, 45–48]. In our study, we did not evaluate specific T cell subtypes in the cornea. However, the significant reduction in ocular infection and pathogenesis in VC2 versus HSV-1(F)-vaccinated mice following McKrae challenge indicates it is possible that the majority of infiltrating T cells in VC2 belongs to the CD8+ phenotype. In support of this hypothesis, CD8+ effector memory cells were expanded in the draining lymph node whereas CD4+ Tem cells remained at lower levels and like those induced by the HSV-1(F) infection. It is not clear whether this CD8+ T cell expansion represent HSV-1-specific responses, since we did not assess viral specific T cell responses. In addition, we did not look at the latent virus in TG to address whether vaccinated animals can actually prevent viral latency. Future studies will include these analysis of vaccine specific T cell responses and viral latency.

In addition to CD8 T cell, anti-HSV antibody had been reported to have beneficial role against HSV-1 infection [18, 19, 49–53]. Recently, two HSV-1 vaccine studies [18, 19] reported the involvement of antibody in conferring protection against ocular challenge. Moreover, human anti-HSV-1 gD specific monoclonal antibody prevented the acquisition of infection in mice [49]. Although pre-challenge HSV-1 specific serum antibody titer was not different among groups, serum antibody response went significantly higher in VC2-vaccinated group followed by the challenge. In addition, pre-challenge neutralization by serum from VC2 vaccinated mice exhibited significantly higher neutralization titers in comparison to serum produced by the HSV-1(F) vaccinated mice. It is likely, that VC2 generates a protecrive immune response at the site of intramuscular infection that results in higher innate and subsequent adoptive immune responses including higher levels of both humoral and cellular responses. These hypotheses are currently under investigation.

## Supporting information

S1 Fig Gating strategy for cellular phenotyping. (TIF)
