# Supplementary figures and images for: Intramuscular vaccination of mice with the human herpes simplex virus type-1(HSV-1) VC2 vaccine, but not its parental strain HSV-1(F) confers full protection against lethal ocular HSV-1 (McKrae) pathogenesis
Source: PLoS One. 2020 Feb 6;15(2):e0228252. doi: 10.1371/journal.pone.0228252 (PMC7004361; doi:10.1371/journal.pone.0228252)

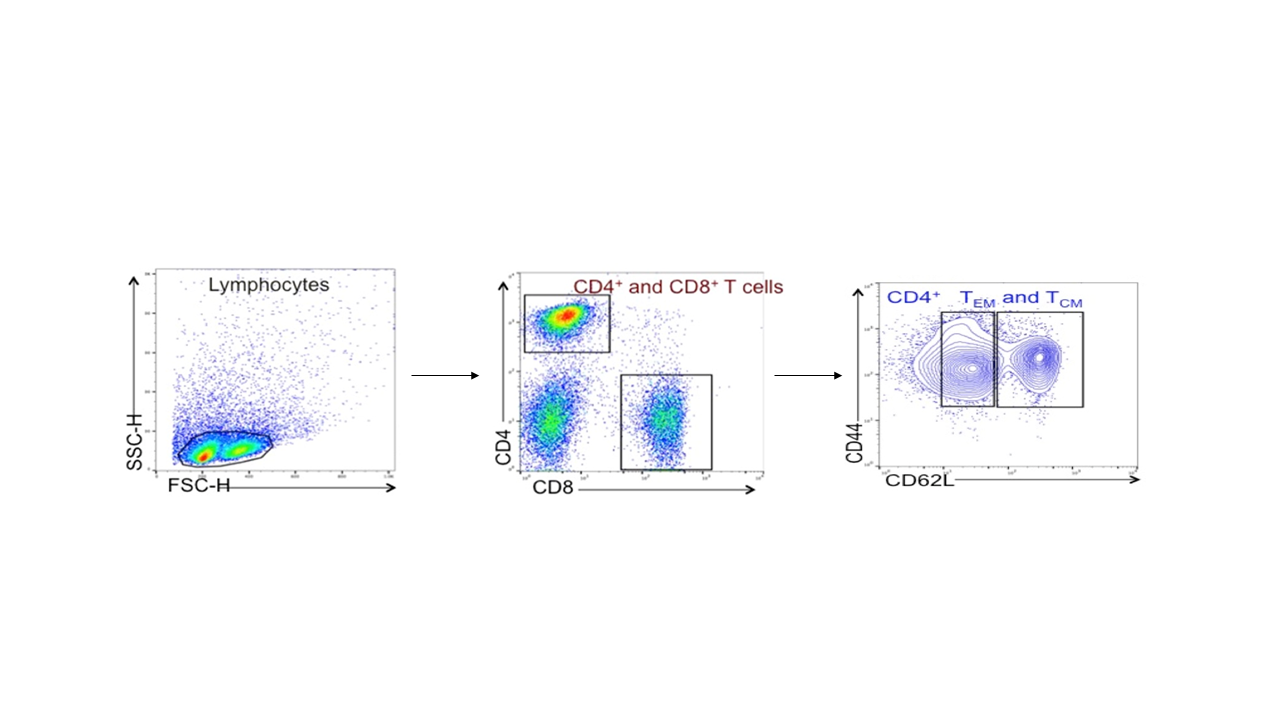

Supplement: S1 Fig — (TIF) [file pone.0228252.s001.tif]
